# Low temperature, mechanical wound, and exogenous salicylic acid (SA) can stimulate the SA signaling molecule as well as its downstream pathway and the formation of fruiting bodies in *Flammulina filiformis*

**DOI:** 10.3389/fmicb.2023.1197498

**Published:** 2023-08-22

**Authors:** Ziyan Li, Jin Wen, Zhuohan Jing, Hui Li, Jiahua Huang, Chengjin Yuan, Lijun Xian, Lingling Gao, Jian Zhu, Baogui Xie, Yongxin Tao

**Affiliations:** ^1^College of Horticulture, Fujian Agriculture and Forestry University, Fuzhou, Fujian, China; ^2^Mycological Research Center, Fujian Agriculture and Forestry University, Fuzhou, Fujian, China; ^3^Institute of Cash Crops, Hebei Academy of Agriculture and Forestry Sciences, Shijiazhuang, China

**Keywords:** *Flammulina filiformis*, low temperature, mechanical wound, salicylic acid, primordia formation

## Abstract

Low temperature (LT) and mechanical wound (MW), as two common physics methods, have been empirically used in production to stimulate the primordia formation of *Flammulina filiformis*, which is typically produced using the industrial production mode. However, the detailed effect on the fruiting body formation and important endogenous hormones and signaling pathways in this process is poorly understood. In this study, LT, MW, their combination, i.e., MW + LT, and low concentration of SA (0.1 mM SA) treatments were applied to the physiologically mature mycelia of *F. filiformis*. The results showed that the primordia under the four treatments began to appear on the 5th−6th days compared with the 12th day in the control (no treatment). The MW + LT treatment produced the largest number of primordia (1,859 per bottle), followed by MW (757), SA (141), and LT (22), compared with 47 per bottle in the control. The HPLC results showed that the average contents of endogenous SA were significantly increased by 1.3 to 2.6 times under four treatments. A total of 11 SA signaling genes were identified in the *F. filiformis* genome, including 4 *NPR* genes (*FfNpr1-4*), 5 *TGA* genes (*FfTga1-5*), and 2 *PR* genes (*FfPr1-2*). *FfNpr3* with complete conserved domains (ANK and BTB/POZ) showed significantly upregulated expression under all four above treatments, while *FfNpr1/2/4* with one domain showed significantly upregulated response expression under the partial treatment of all four treatments. *FfTga1-5* and *FfPr1-2* showed 1.6-fold to 8.5-fold significant upregulation with varying degrees in response to four treatments. The results suggested that there was a correlation between “low temperature/mechanical wound—SA signal—fruiting body formation”, and it will help researchers to understand the role of SA hormone and SA signaling pathway genes in the formation of fruiting bodies in fungi.

## Introduction

*Flammulina filiformis* is a common and popular edible mushroom in East Asia and has a delicious taste and high nutritional value (Yi et al., 2013). It is also called “wisdom-enhancing mushroom” because it is rich in protein, amino acids, and mineral content, especially lysine, which cannot be synthesized by the human body (Liu et al., 2019). In addition, it also contains quite a few biologically active proteins, polypeptides, polysaccharides, β-glucans, sterols, ergothioneine, dilinolein acid, and hemisceramide, among others (Cai et al., 2013). *F. filiformis* ranks fourth in the production scale among the edible mushrooms and ranks first among the industrially cultivated edible fungi in China (Li and Xu, 2022). At present, it is produced using the mechanized and automated industrial production mode. The quality and time of fruiting body formation, especially *F. filiformis*, determine the economic benefits of the edible mushroom factories.

*F. filiformis* is a fascicled mushroom, and in each cultivation bottle, it is produced by clustering together many individual and elongated fruiting bodies. The number of fruiting bodies or primordia (the most initial form of fruiting bodies) in each cultivation bottle directly determines its yield, while the time of fruiting body formation is related to the production cycle, which is associated with the consumption of energy and the utilization rate of the mushroom cultivation room. Fruiting body formation is a process of transition from the vegetative stage to the reproductive stage, which is regulated by both external environmental factors (light, temperature, and air) and intracellular signals and genes (endogenous hormones, signaling molecules, and their pathway genes) (Sakamoto et al., 2004; Hao et al., 2019). Therefore, it is very important to explore the effective environmental factors and related intracellular signal transduction and gene expression during the fruiting body formation, which is also focused as a popular topic in the development studies of fungi.

Previously, it has been reported that several factors can induce fruiting body formation in edible fungi. In *Lentinus edodes*, the fruiting body formation can be induced by blue light (460–475 nm) and low temperature (18°C) (Nakazawa et al., 2008). In *Agaricus bisporus*, the primordia could be initiated by air temperature cooldown (from 25°C to 18°C) and ventilation for gas release from the culture chamber (Eastwood et al., 2013). In the *F. filiformis* factory, before the formation of primordia, the surface of the cultivation substrate covered with *F. filiformis* mycelia often needs to be scraped off with a mechanical knife (which is called “scratching hypha” in production) (Lu et al., 2016; Ho and Suzuki, 2019). However, the detailed effect on primordia formation for these environmental conditions is lacking, and the mechanism by which these measures or conditions promote primordia formation remains unclear.

Studies related to fruiting body formation have been reported on several genes in fungi. For example, the inactivation of *wc-1* and *wc-2* (two photoreceptor encoding genes) abolished fruiting body formation in *Schizophyllum commune* (Ohm et al., 2013). Similar blind phenotypes in fruiting body formation were also observed in *dst1* and *dst2* (*wc-1* and *wc-2* homologs) deletion mutants in *Coprinopsis cinerea* (Kamada et al., 2010). The *priB* gene was found to be most abundantly transcribed in primordia and in the early stages of fruiting body formation in *Lentinula edodes* (Miyazaki et al., 2004). The over-expression of the *c2h2* ortholog of *Agaricus bisporus* could accelerate the formation and development of fruiting bodies (Pelkmans et al., 2016). In *F. filiformis*, a hydrophobin gene, *Hyd9*-silenced transformants, exhibited sparse aerial hyphae and resulted in a decrease in the number of primordia (Tao et al., 2019). However, little is known about how these genes mentioned above (except photoreceptors) are signaled and regulated by external factors. With regard to the intracellular hormone or signaling molecule, there have been few studies about them during the fruiting body formation in fungi.

In plants, many studies have shown that when subjected to external stress such as cold, drought, pathogen infecting, insect biting, or tissue damage, the intracellular salicylic acid (SA) signal pathway was rapidly activated and produced in large amount (Seo et al., 2007; Khan et al., 2015; Mostafa et al., 2022; Zhang et al., 2023). SA as a hormone plays a critical role in the regulation of a multitude of physiological processes such as seed germination, vegetative growth, photosynthesis, respiration, thermogenesis, flower formation, seed production, responding to pathogens, and stimulating plant defense immunity (Lastochkina et al., 2020; Ansari et al., 2021). Under biotic stress conditions, SA fulfills a key function as an endogenous signal mediating local defense responses (Bauer et al., 2022; Zhang et al., 2023). In fungi, it has been found that high temperatures could increase the SA content in the extracellular fluid of *Pleurotus ostreatus* (Qiu et al., 2018). Therefore, it is reasonable to speculate whether the changes in intracellular SA and its downstream pathway would also be activated when edible fungi mycelia are subjected to moderate stress including low temperature and mechanical wound (such as “scratching hypha”).

The current understanding of SA hormone and SA-dependent signal transduction pathways is very limited in fungi. In plants, it has been well studied that non-expressor of pathogenesis-related proteins (NPRs) are the effector of SA signaling molecules. The basic model of the SA signaling pathway is that SA accumulation stimulates the translocation of NPR into the nucleus, where it interacts with the TGA transcription factor family members (a subgroup of the basic leucine zipper transcription factor family) and enhances the binding of these factors to SA response elements in the promoters of pathogenesis-related genes (*PR* genes), and thus ultimately activates the expression of *PR* genes and causes systemic acquired resistance (SAR) (Rushton and Somssich, 1998; Kesarwani et al., 2007). The promoters of *PR* genes generally contain an activator sequence-1 (as-1-like elements), which is important for SA-inducible gene expression (Durrant and Dong, 2004).

In this study, the explicit effect on the fruiting body formation of four exogenous factors including low temperature, mechanical wound, the combination of mechanical wound and low temperature, and exogenous SA in *F. filiformis* was studied. The changes in endogenous SA content and SA signaling pathway genes were detected under these conditions. The results of this study will help researchers to understand the interrelationship between “low temperature/mechanical wound—SA signal—fruiting body formation” in fungi.

## Materials and methods

### Strains and culture conditions

*Flammulina filiformis* dikaryotic strain FL19 (hybridized from monokaryon strains L11 and L22) is obtained from the Fujian Edible Fungi Germplasm Resource Collection Center of China. The strain was cultivated regularly in potato dextrose agar (PDA) at 25°C. To screen for the suitable concentration of exogenous SA, three different concentrations of SA (0.1 mM, 0.5 mM, and 2.0 mM) were added to the PDA medium to cultivate the *F. filiformis* mycelia, and the control was without the addition of exogenous SA. To explore the effect of different treatments on the fruiting body formation, an equal amount of the PDA strain of FL19 was inoculated into the cultivation medium that is 49% sawdust, 30% cottonseed hull, 20% wheat bran, and 1% pulverized lime, adding water to 60% water content (Li et al., 2022).

Cultivation bottles after inoculation were placed at 22°C for culture. After ~20 days, the inside and outside of the cultivation substrate in the bottles were covered with mycelia of *F. filiformis*. Then, the cultivation bottles filled with mycelia with the same growth status and age were selected for the following treatments. The other cultivation bottles without any treatment (only opened the bottle caps and continued to place at 22°C) were used as the control (CT). There were four treatments as follows: (1) mechanical wound (MW), the cultivation bottles were treated by “scratching hypha” (the standard practice in production is that the surface of cultivation substrate covered with *F. filiformis* mycelia was scraped off ~1 cm by a mechanical knife) and placed at 22°C; (2) low temperature (LT), the cultivation bottles without “scratching hypha” treatment were placed at 16°C; (3) the combination of MW and LT, the cultivation bottles with “scratching hypha” treatment were placed at 16°C; (4) 0.1 mM SA treatment, the cultivation bottles without “scratching hypha” treatment were placed at 22°C and were sprayed by 0.1 mM SA once a day for a total of three times. We observed the mycelia surface every day and counted the days of the beginning of primordia formation and the end of total primordia formation under each treatment. The changes in mycelia and the primordia formation were photographed and recorded every 3 days.

### Determination of SA content

The samples of mycelia growing on the surface of the substrate under four treatments and the control were collected at 6 h after treatments, referring to the results of the study by Schaller et al. (2000). According to the method of Szkop et al. (2016), pre-sample preparation is as follows: 0.5 g fresh mycelia were successively added to 1.5 ml 0.4 M K_2_HPO_4_ (3.48 g water was added to 50 ml) to grind, and after strong vortexing for 1 min and incubation in a water bath at 70°C for 15 min, strong vortexing was continued for 1 min and centrifugation was performed at 16,000 × g for 10 min. In total, 1 ml of the supernatant was transferred to a new 2 ml centrifuge tube, 150 μl of 10 M HCl was added and shaken 6–8 times, the acidified supernatant was extracted with 800 μl of ethyl acetate, and the mixture was vortexed to make it fully mixed. After centrifugation at 16,000 × g for 10 min, 600 μl of the upper organic phase was removed and transferred to a new centrifuge tube, and 600 μl of 0.2 M PBS was added. The mixed liquid was vortexed vigorously for 1 min and centrifuged at 16,000 × g for 1 min. Finally, 500 μl of the lower phase was transferred to the light-protected HPLC bottle until the next direct analysis. All reagents were used on the spot, and the prepared samples were not prepared overnight.

Analysis of equipment and chromatographic conditions are as follows: According to the method of Szkop et al. (2016), the analysis equipment was DGU-20A3 (Shimadzu, Japan), including a binary pump, a UV detector, an autosampler, and external equipment for data acquisition and integration. The chromatographic column was InertSustain C18 (Shimadzu Japan), was filled with octadecylsilane, and has a larger surface area. Phase A was 60 mM phosphate-buffered saline (PBS), phase B was 80% acetonitrile, and the linear elution procedure was used (0–10 min is 10% phase B, 10–13 min is 30% phase B, 13–22 min is 100% phase B, and 22–27 min is 10% phase B), the column temperature was 34°C, the flow rate was 1 ml/min, and the injection volume was 20 μl.

### Identification and analysis of SA signaling pathway genes in *F. filiformis*

The SA signal pathway genes of *Arabidopsis thaliana* were obtained by searching the SA signal pathway by KEGG. According to the protein sequences of the *NPR, TGA*, and *PR* genes that have been identified in *Arabidopsis*, their respective homologous genes in the L11 genome of *F. filiformis* were obtained by the local blast search. The gene structure is analyzed by SnapGene V5.2, and the conservative domain is predicted by InterProScan online software (http://www.ebi.ac.uk/Tools/pfa/iprscan/), and the nuclear localization was predicted at Cell-PLoc 2.0. The gene-protein structure diagram was drawn on the Exon-Intron Graphic Maker website (http://www.wormweb.org/exonintron). To construct the phylogenetic tree of all NPR/TGA/PR proteins, full-length protein sequences were aligned using Clustal Omega (Sievers et al., 2014). The phylogenetic tree was constructed using the MEGA, and developmental relationships were analyzed using the neighbor-joining (NJ) method and the ultrafast bootstrap approximation approach with 1000 replicates (Evans et al., 2006).

### Total RNA extraction and RT-qPCR

The samples of mycelia growing on the surface of the substrate under four treatments and the control were collected 3 h after the treatments (referring to the results of Han et al., 2022) for the determination of transcriptional levels of SA signaling pathway genes by RT-qPCR. All the samples were collected in equal mass (0.5 g) and frozen in liquid nitrogen for RNA extraction. Total RNA was extracted according to the standard method described in the instructions of the Omega E.Z.N.A. plant RNA kit (Omega Bio-Tek, USA). When the A260/A280 ratio of RNA is 2.0 to 2.1 (measured using Thermo Science NanoDrop Lite; USA) and the concentration is diluted to 500 ng/μl, it is used for cDNA synthesis. The cDNA was synthesized by reverse transcription using the TransScript All-in-One First-Strand cDNA Synthesis SuperMix for qPCR kit (TransGen, China). Three internal reference genes stably expressed in *F. filiformis* (*RNB, V-ATP*, and β*-TUB*) were used for the standardization of RT-qPCR (Yang et al., 2022). All the primers for RT-qPCR are shown in Supplementary Table S1. The RT-qPCR was performed according to the standard method described in the instructions of *PerfectStart* Uni RT and qPCR Kit (TransGen Biotech, Beijing). The relative gene expression levels were determined according to the 2^−Δ*ΔCt*^ method.

### Statistical analysis

In this study, all the results were carried out with three independent biological replicates to ensure that the trends and relationships observed were reproducible. The error bars indicate the standard deviation (SD) from the mean of triplicate samples. The significance of the data was analyzed by using one-way ANOVA and multiple comparison tests.

## Results

### Effects of four treatments including LT, MW, MW + LT, and exogenous SA on the primordia formation in *F. filiformis*

**Figure 1:**
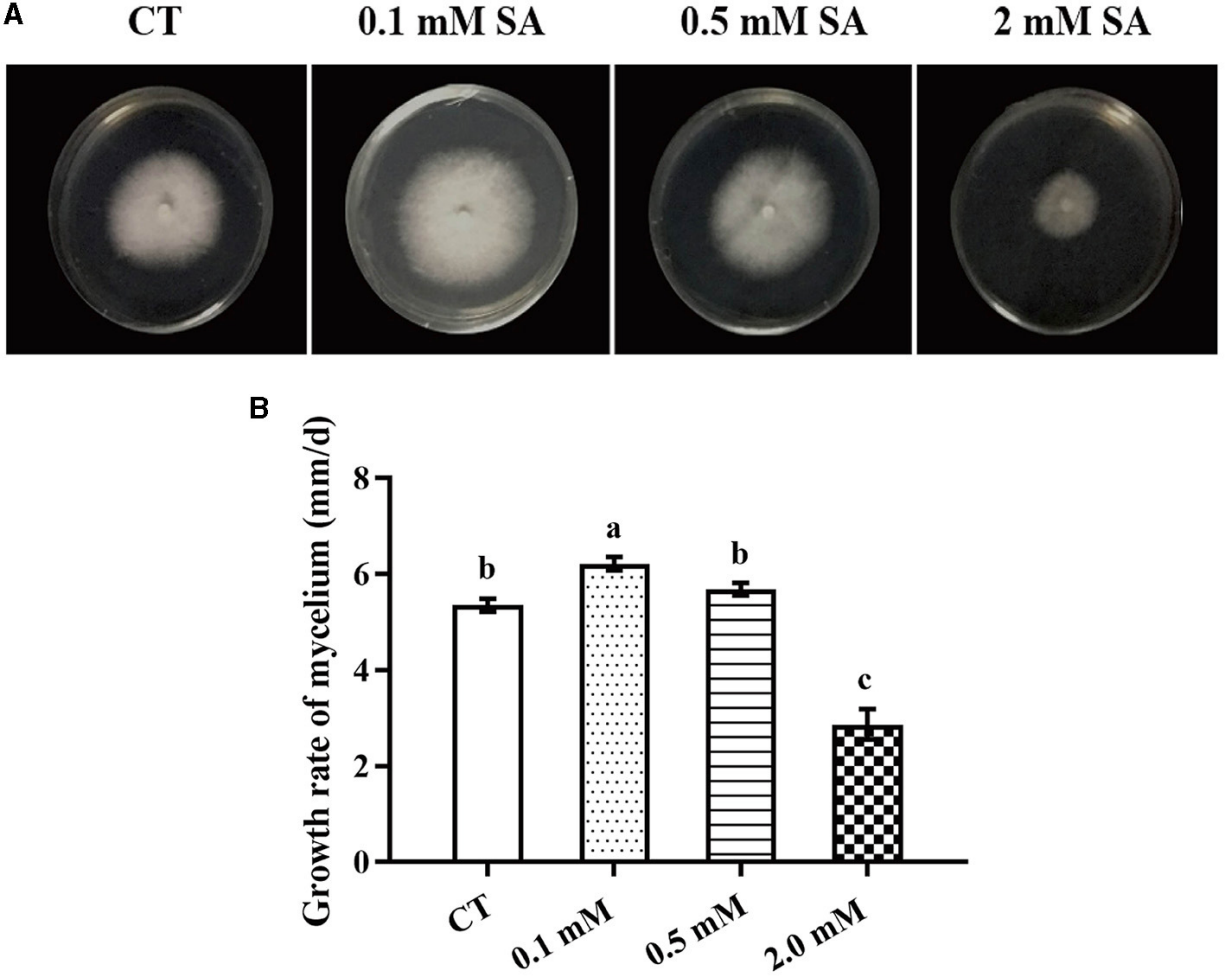
Morphology and growth rate of *F. filiformis* mycelia under different concentrations of SA. **(A)** Colony morphology of *F. filiformis* under different concentrations of SA (0.1 mM, 0.5 mM, and 2 mM). CT: control (0 mM SA). **(B)** The growth rate of *F. filiformis* mycelia under different concentrations of SA. CT: control (0 mM SA). Different lowercase letters indicate significant differences at *p* < 0.05.

To identify the factors that significantly affect the primordia formation in *F. filiformis*, three treatments, namely LT, MW, and MW + LT, were designed according to the usual measures used in *F. filiformis* factories. Meanwhile, the exogenous SA was also used as a treatment. First, we screened the suitable concentration of exogenous SA for *F. filiformis* mycelia. Compared with the control (adding 0 mM SA), the growth rate of mycelia under 0.1 mM SA treatment was increased by 16.3%, while that under 0.5 mM SA treatment showed no significant difference with the control (Figures 1A, B). On the contrary, the growth rate of mycelia under 2.0 mM SA treatment was decreased by 49.5% (Figure 1B). The results showed that 0.1 mM SA treatment (low concentration) could promote mycelial growth, but 2.0 mM SA treatment (high concentration) could seriously inhibit mycelial growth. Therefore, the 0.1 mM SA treatment was used to perform the follow-up experiment because of its dosage of lower concentration and the promoting effect on mycelial growth.

**Figure 2:**
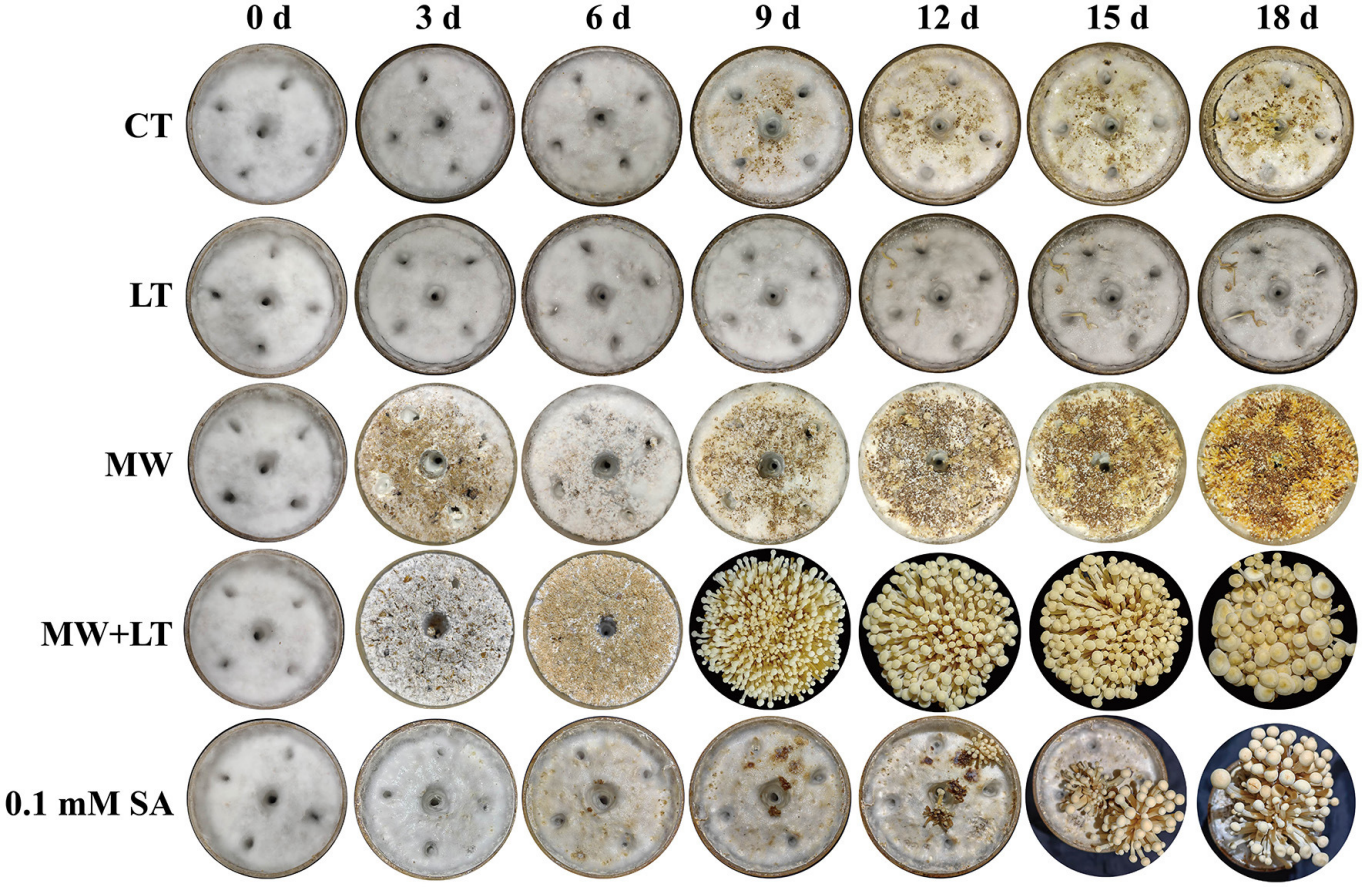
Effects of four treatments, namely LT, MW, MW + LT, and 0.1 mM exogenous SA, on the colony morphology and primordia formation in *F. filiformis*. CT: Control (no treatment), LT, low temperature; MW, mechanical wound; MW + LT, the combination of mechanical wound and low temperature, 0~18 d: 0 to 18 days after four treatments.

**Figure 3:**
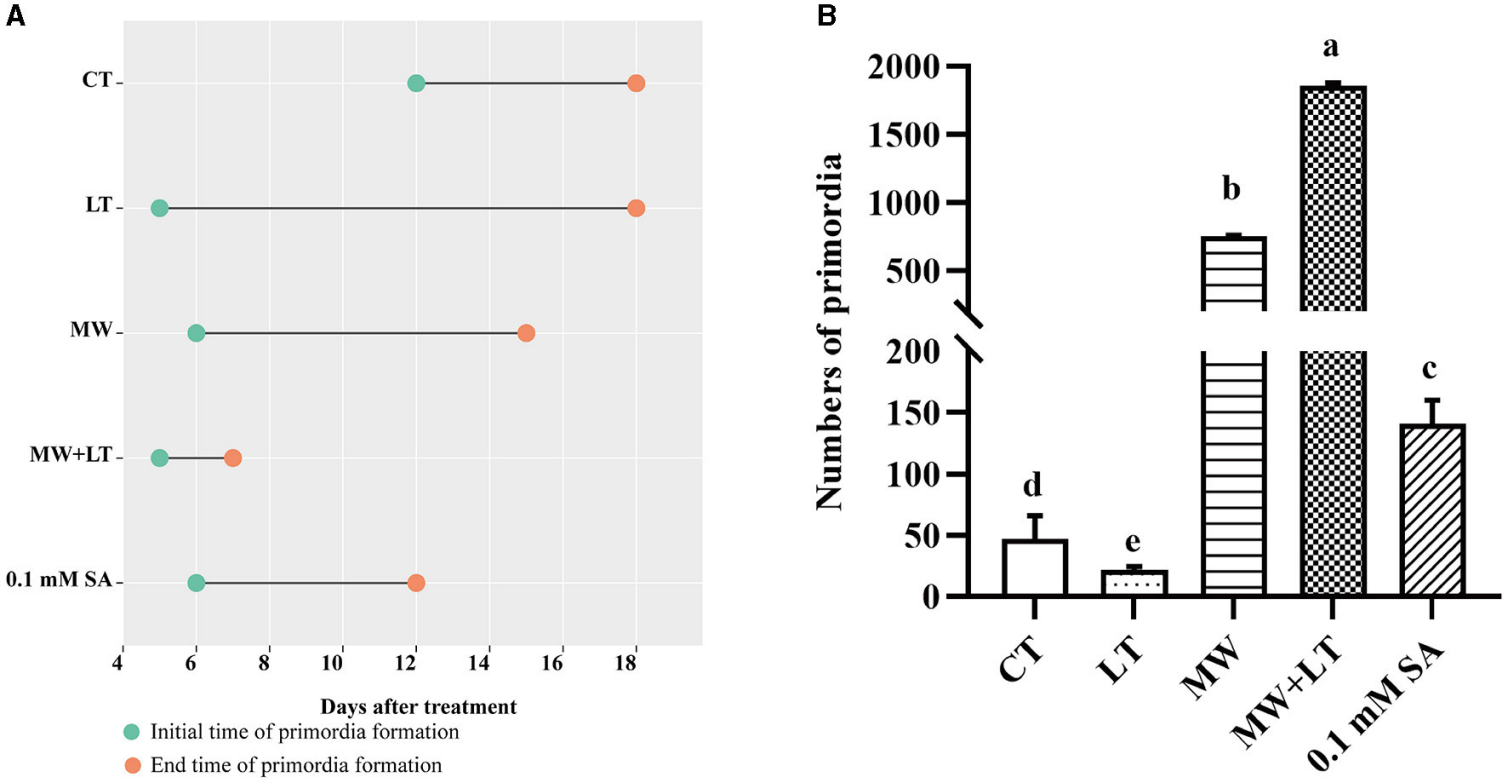
Effects of four treatments, namely LT, MW, MW + LT, and 0.1 mM exogenous SA, on the time and amount of primordia formation in *F. filiformis*. CT, Control (no treatment); LT, low temperature; MW, mechanical wound; MW + LT, the combination of mechanical wound and low temperature. **(A)** The initial and end time of *F. filiformis* primordia formation under four treatments. **(B)** The number of *F. filiformis* primordia formation under four treatments. Different lowercase letters indicate significant differences at *p* < 0.05.

All treatments including the control (no treatment) grew out the primordia during 18 days but varied dramatically in time and quantity (Figure 2). The primordia under the LT, MW, MW + LT, and 0.1 mM SA treatments began to appear on the 5th, 6th, 5th, and 6th days, respectively, while the time of initial appearance under no treatment was 12 days (Figure 3A). The time span of primordia formation is also different among the different conditions. The primordia formation under the MW + LT condition lasted for 3 days (shortest), while the period of primordia formation under the LT, MW, 0.1 mM SA, and no treatment conditions lasted for 14, 10, 7, and 7 days, respectively. Moreover, we counted the numbers of primordia and found that the MW + LT treatment grew the largest number of primordia, and the average number is 1,859 per bottle. The smallest number of primordia was under LT treatment, and its average number is 22 per bottle, followed by the control (no treatment, with 47 per bottle). The average numbers of primordia under MW and 0.1 mM SA treatments were 757 and 141 per bottle, respectively (Figure 3B).

### The content change of endogenous SA under four treatments including LT, MW, MW + LT, and exogenous SA in *F. filiformis*

**Figure 4:**
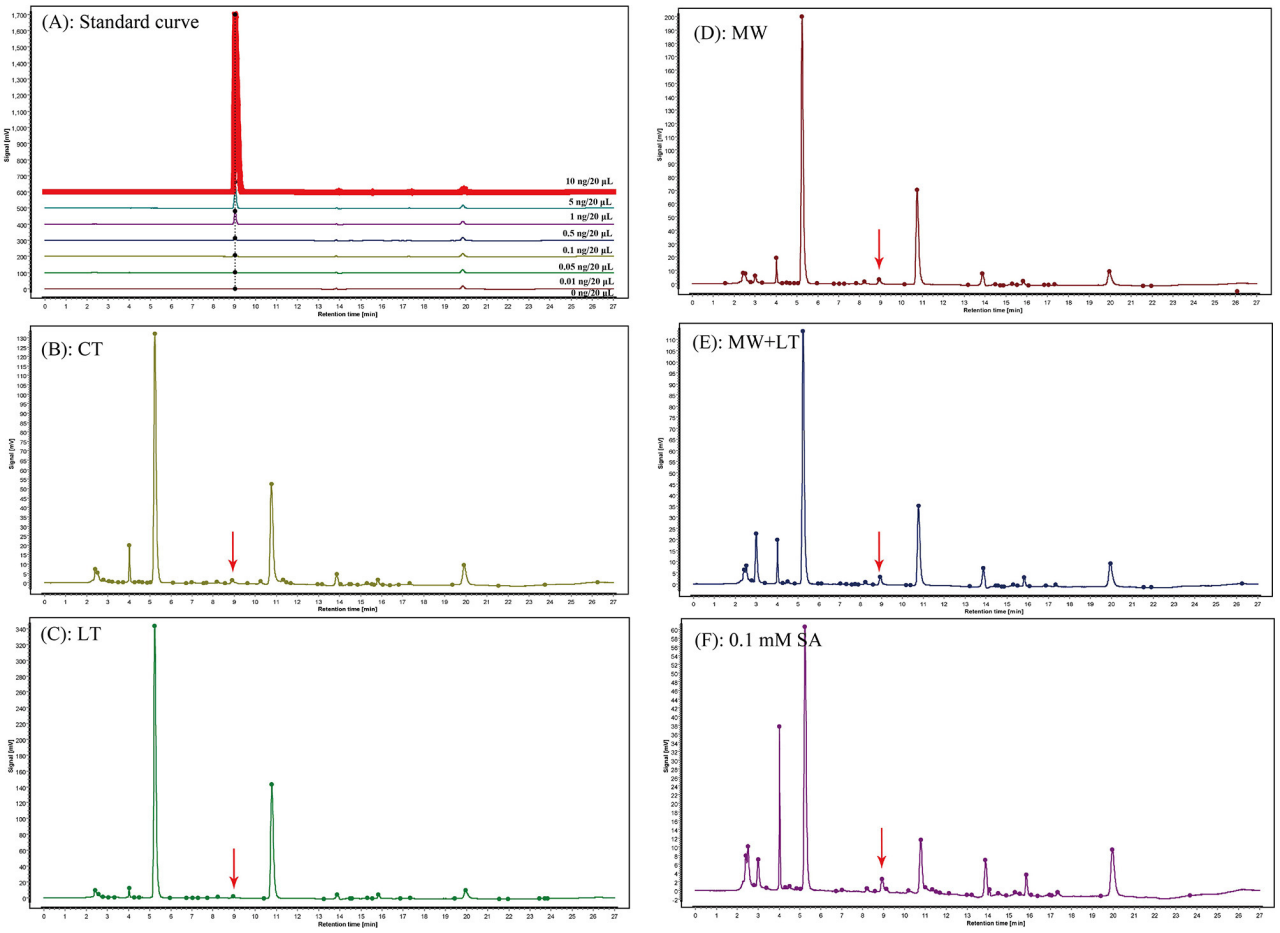
Peak charts of SA determined by HPLC under the control and four treatments including LT, MW, MW + LT, and 0.1 mM SA. CT, Control (no treatment); LT, low temperature; MW, mechanical wound; MW + LT, the combination of mechanical wound and low temperature. **(A)** Standard peak charts of seven standard samples with gradient SA concentrations (the concentration from low to high was 0, 0.01, 0.05, 0.1, 0.5, 1, 5, and 10 ng/20 μl, respectively). **(B–F)** Peak charts of SA determined by HPLC under the control and four treatments, namely LT, MW, MW + LT, and 0.1 mM SA. The retention time and absorption peak of SA in the samples were marked by the red arrow.

**Figure 5:**
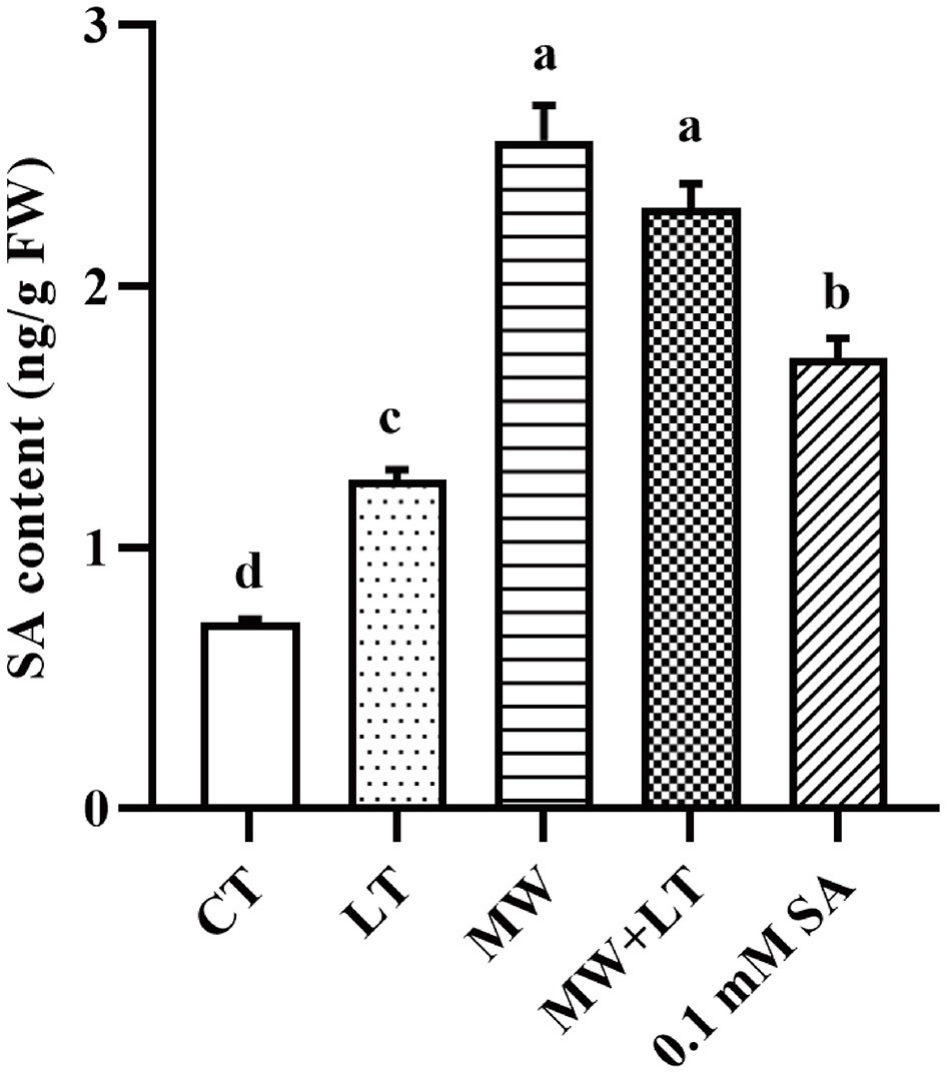
Content change of endogenous SA under four treatments, namely LT, MW, MW + LT, and 0.1 mM exogenous SA in *F. filiformis*. Different lowercase letters indicate significant differences at a *p-*value *of* < 0.05.

The contents of endogenous SA under four treatments (LT, MW, MW + LT, and 0.1 mM SA) were detected using HPLC. The peak plots of the standard curve of SA with seven concentration gradients (0, 0.01, 0.05, 0.1, 0.5, 1, 5, and 10 ng/20 μl), and the peak plots of SA with four treatments are clearly shown in Figure 4. The standard curve for salicylic acid was formulated as Y = 1251279.9X + 4775.8, R^2^ = 0.9999. The HPLC results showed that the average content of endogenous SA in the control sample was 0.7 ng/g, and it was increased to 1.3-, 2.6-, 2.3-, and 1.7-fold under LT, MW, MW + LT, and 0.1 mM exogenous SA treatments as compared with control, respectively (Figure 5). It is clear that there was a significant increase in endogenous SA content under all four treatments, and among them, the MW and MW + LT treatments (no significant difference between them) induced the greatest increase of SA in a short time, followed by 0.1 mM exogenous SA spraying.

### Identification of SA signal pathway genes in *F. filiformis*

**Figure 6:**
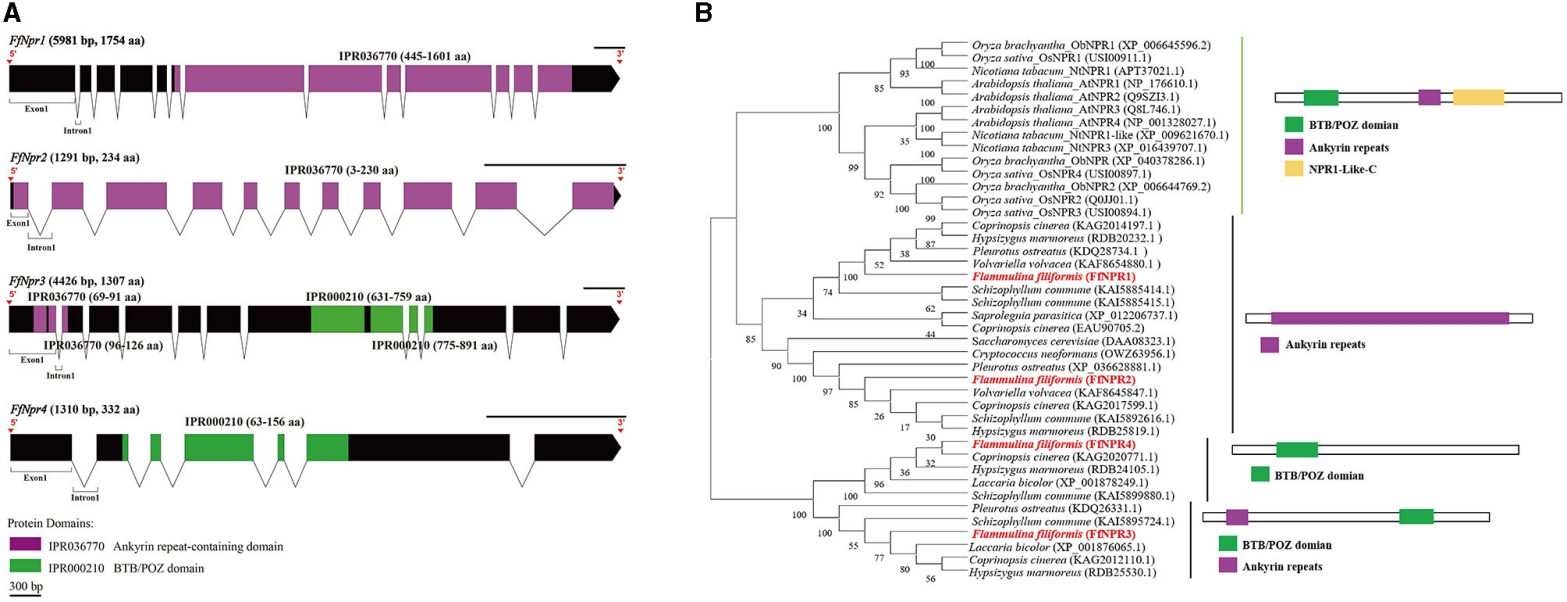
Gene structures of *FfNpr* family genes in *F. filiformis* and phylogenetic tree analysis of NPR proteins. **(A)** Gene structures and protein conserved domains of the *FfNpr1/2/3/4* in *F. filiformis*. The gaps indicate introns, non-gaps indicate exons, different colors indicate different domains, and the scale bar is 300 bp. The same module color represents the same type of domain. **(B)** Phylogenetic tree of FfNPR1/2/3/4 and NPR proteins from other species including fungi and plants. The bold red font represents NPR in *F. filiformis*.

**Figure 7:**
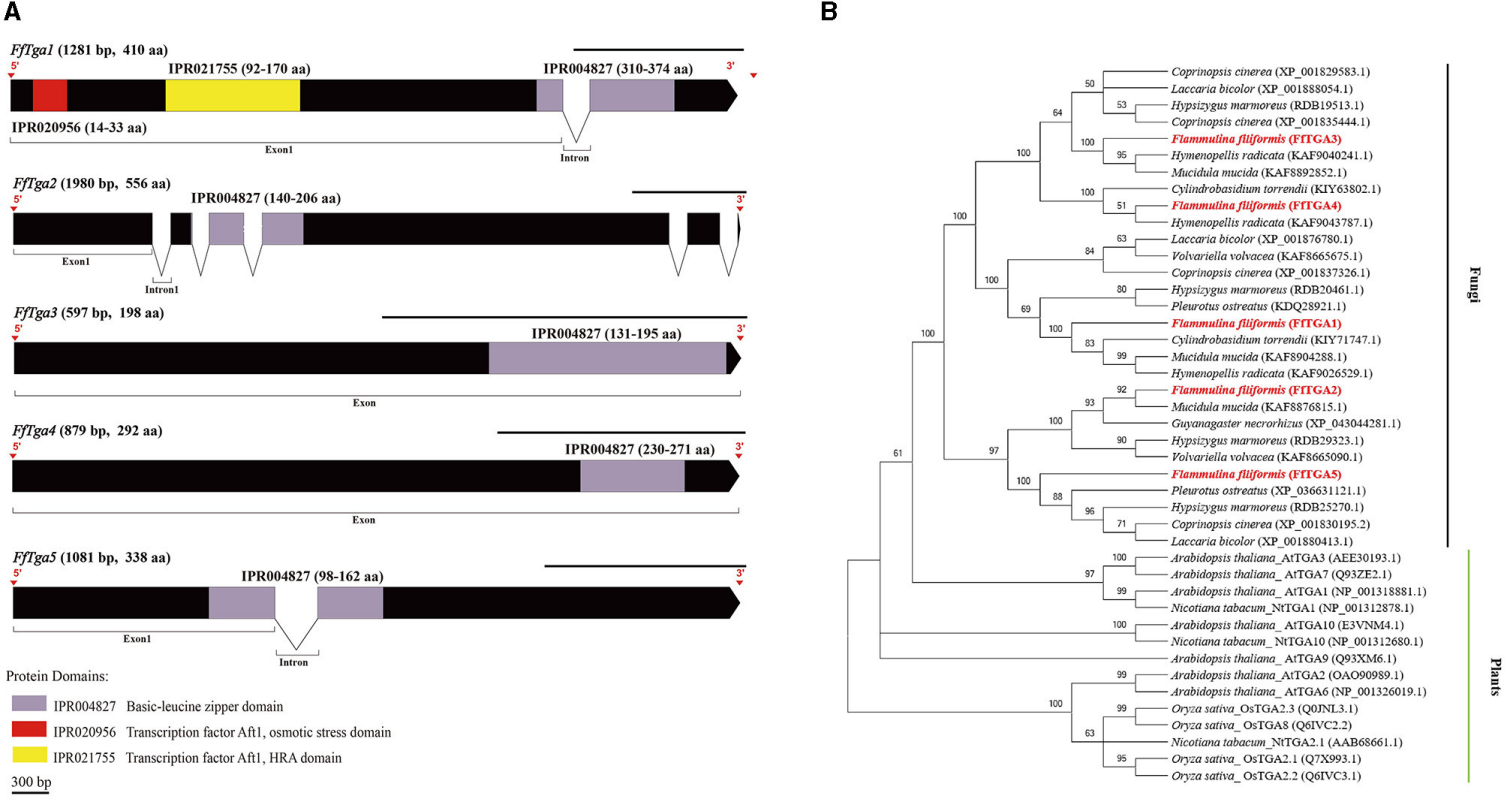
Gene structures of *FTga* family genes in *F. filiformis* and phylogenetic tree analysis of TGA proteins. **(A)** Gene structures and protein conserved domains of the *FfTga1/2/3/4/5* in *F. filiformis*. The gaps indicate introns, non-gaps indicate exons, different colors indicate different domains, and the scale bar is 300 bp. The same module color represents the same type of domain. **(B)** Phylogenetic tree of FfTGA1/2/3/4/5 and TGA proteins from other species including fungi and plants. The bold red font represents TGA in *F. filiformis*.

**Figure 8:**
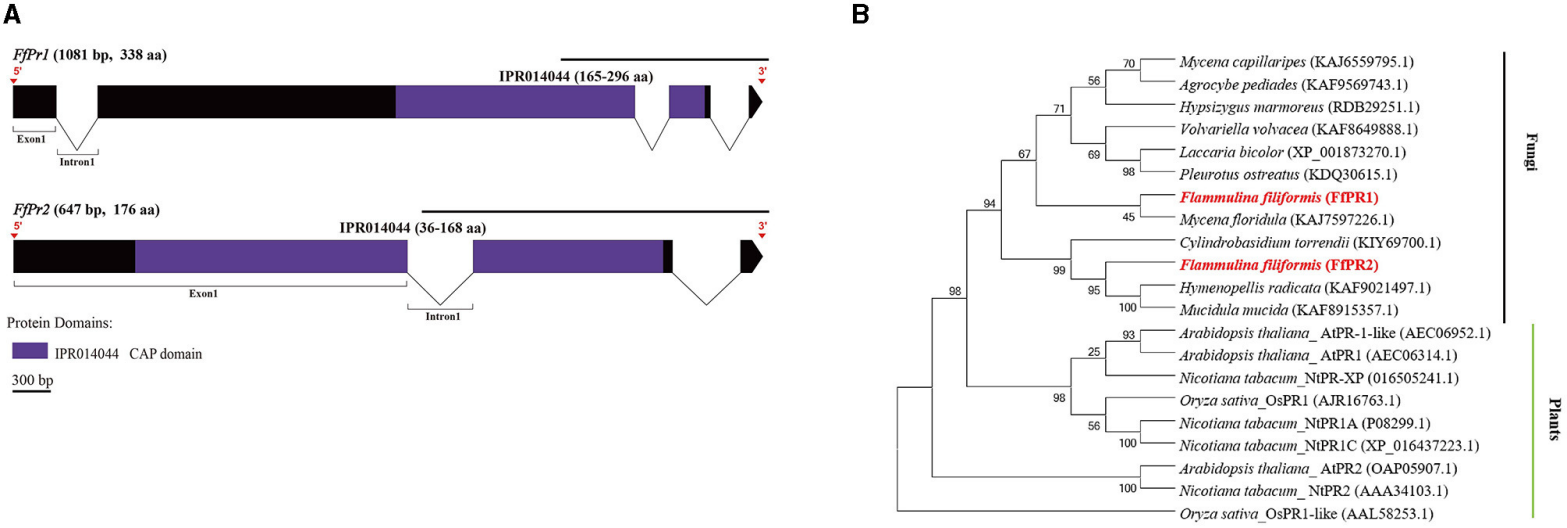
Gene structures of *FfPr* family genes in *F. filiformis* and phylogenetic tree analysis of PR proteins. **(A)** Gene structures and protein conserved domains of the *FfPr1/2* in *F. filiformis*. The gaps indicate introns, non-gaps indicate exons, different colors indicate different domains, and the scale bar is 300 bp. The same module color represents the same type of domain. **(B)** Phylogenetic tree of FfPR1/2 and PR proteins from other species including fungi and plants. The bold red font represents PR in *F. filiformis*.

A total of 11 homologous genes of SA signal pathway genes were obtained from L11 genome, including four *NPR* family genes (*FfNpr1, FfNpr2, FfNpr3*, and *FfNpr4*), five *TGA* family genes (*FfTga1, FfTga2, FfTga3, FfTga4*, and *FfTga5*), and two *PR* family genes (*FfPr1* and *FfPr2*) with the GenBank ID numbers, as shown in Table 1. Moreover, the lengths and structures of these genes are shown in Figures 6A, 7A, 8A; detailed information including the numbers of exons and introns is shown in Supplementary Table S2.

**Table 1:** SA signaling pathway genes in *F. filiformis*.

**Family gene name**	**Gene symbol**	**Conserved domain**	**Accession number in *F. filiformis***
Non-expression of pathogenesis-related genes	*FfNpr1*	Ankyrin repeat-containing (IPR036770)	OQ679972
	*FfNpr2*	Ankyrin repeat-containing (IPR036770)	OQ679973
	*FfNpr3*	Ankyrin repeat-containing (IPR036770);	OQ679974
		BTB/POZ (IPR00210)	
	*FfNpr4*	BTB/POZ (IPR00210)	OQ679975
TGACG-binding factors	*FfTga1*	Basic leucine zipper (IPR004827); Transcription factor *Aft1*, osmotic stress (IPR020956); Transcription factor *Aft*, HRA (IPR021755)	OQ679976
	*FfTga2*	Basic leucine zipper (IPR004827)	OQ679977
	*FfTga3*	Basic leucine zipper (IPR004827)	OQ679978
	*FfTga4*	Basic leucine zipper (IPR004827)	OQ679979
	*FfTga5*	Basic leucine zipper (IPR004827)	OQ679980
Pathogenesis-related genes	*FfPr1*	CAP (IPR014044)	OQ679981
	*FfPr2*	CAP (IPR014044)	OQ379982

*F. filiformis FfNpr1* (the length of gDNA is 5981 bp), *FfNpr2* (1291 bp), *FfNpr3* (4426 bp), and *FfNpr4* (1310 bp) encode proteins with lengths of 1754 aa, 234 aa, 1307 aa, and 332 aa, respectively (Figure 6A). The NPR proteins in plants typically contain three conserved domains: BTB/POZ domain, Ankyrin repeats, and NPR1-Like-C. The conserved domain analysis showed that FfNPR1/2 contained one type of domain (Ankyrin repeat-containing domain) and FfNPR4 contained another type of domain (BTB/POZ domain), while only FfNPR3 contained both Ankyrin repeat-containing and BTB/POZ domains (Figure 6A). Phylogenetic tree results showed that *Arabidopsis, Nicotiana tabacum, O. sativa*, and *O. brachyantha* clustered in the plant clade, and the NPR proteins of these species simultaneously contain three types of domains. The NPR proteins in fungi can be classified into three distinct types: the first type of NPR contains only Ankyrin repeats domain, including FfNPR1 and FfNPR2; the second type of NPR contains only BTB/POZ domains, including FfNPR4; and the third type of NPR contains both Ankyrin repeat domains and BTB/POZ domains, including FfNPR3 (Figure 6B). NPR proteins from other fungi are present in each type (a systematic naming of NPR family proteins has not been reported in fungal species), suggesting that these three types of NPR proteins are widely distributed in fungi apart from *F. filiformis*.

*F. filiformis FfTga1* (the length of gDNA is 1281 bp), *FfTga2* (1980 bp), *FfTga3* (597 bp), *FfTga4* (879 bp), and *FfTga5* (1081 bp) encode proteins with lengths of 410 aa, 556 aa, 198 aa, 292 aa, and 338 aa, respectively. FfTGA1/2/3/4/5 all belong to the TGA-type subgroup of the basic leucine zipper transcription factor family (IPR004827). In addition, FfTGA1 also contained one Aft1-osmotic stress domain and one Aft1-HRA domain (Figure 7A). Phylogenetic tree results showed that all the TGA proteins of fungi clustered into one cluster and divided into three branches. FfTGA1, as a type with three domains, was clustered into the same clade with its homologs in *Pleurotus ostreatus, Cylindrobasidium torrendii, Mucidula mucida*, and *Hymenopellis radicata*. FfTGA2 and FfTGA5, which have introns and one basic leucine zipper domain as the second type, were clustered into the same clade with its homologs in *M. mucida* and *P. ostreatus*. FfTGA3 and FfTGA4 which have no intron were clustered into the same clade with its homologs in *H. radicata* and *C. torrendii* (Figure 7B).

*F. filiformis FfPr1* (the length of gDNA is 1081 bp) and *FfPr2* (647 bp) encode proteins of 338 aa and 176 aa, respectively. Both FfPR1 and FfPR2 contain a highly conserved CAP domain (Figure 8A), which is called cysteine-rich secretory protein, antigen 5, and pathogenicity-associated protein. Phylogenetic tree relationships showed that FfPR1 and FfPR2 were clustered into the same fungal clade with homologs in *H. radicata, C. torrendii, Mycena floridula, M. mucida*, and other species of fungi (Figure 8B).

### Bioinformatics analysis of SA signal pathway genes of *F. filiformis*

**Figure 9:**
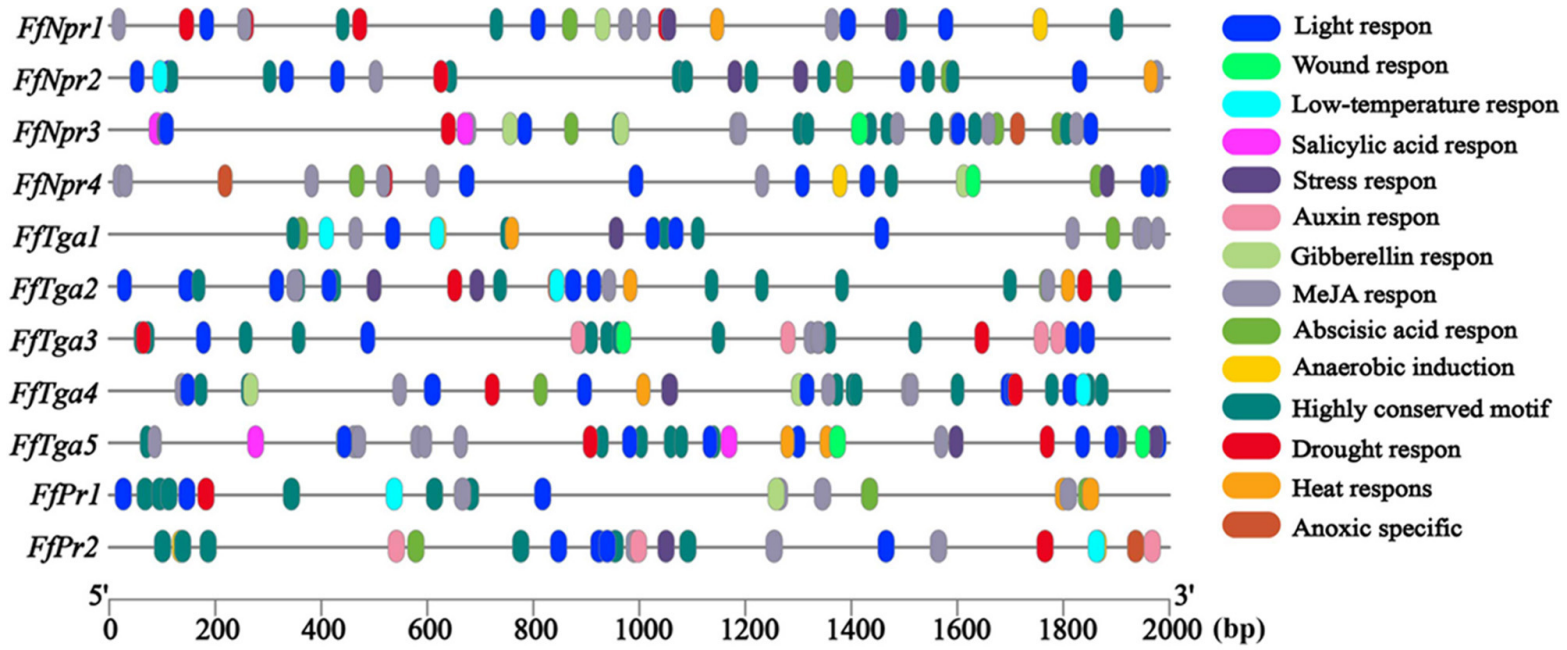
Cis-element analysis of the promoters of SA signaling pathway genes (*FfNpr, FfTga, and FfPr*) in *F. filiformis*. The different color modules represent different cis elements.

To explore the possible factors to which SA signal pathway genes respond, the cis-elements were predicted in the promoter sequences of the *FfNpr, FfTga*, and *FfPr* genes of *F. filiformis*. As shown in Figure 9, several cis-elements related to the response to biotic and abiotic stresses, such as low-temperature response element (LTR, CCGAAA), wound response element (WUN-motif, CAATTACAT), and drought response element (CCAAT-box, CAACGG), were found in the promoters of all 11 genes. The W-box (TTGAC/TTGACC) as a cluster of SA-induced DNA-binding sites was found in the promoters of *FfNpr1/2/3/4*. The cis-regulatory element activation sequence-1 (as-1)-like element motif (TCACG) has been found in the promoter of both *FfPr1* and *FfPr2*, which is essential for the expression of *PR* mediated by SA. The types, numbers, and distribution sites of these cis-elements greatly varied among the promoters of different members of the *FfNpr* family, implying that they have different transcription patterns and regulatory mechanisms in response to abiotic/biotic stress. In addition, the nuclear localization signal (NLS) prediction showed that FfNPR1 has an NLS site which is located at 336–357 aa and FfNPR3 has two NLS sites which are located at 811–827 aa and 1270–1302 aa, while FfNPR2/4 has no obvious NLS prediction. The localization prediction results showed that FfTGA1/2/3/4/5 is also located in the nucleus.

### Transcriptional changes of SA signal pathway genes in response to LT, MW, MW + LT, and exogenous SA

**Figure 10:**
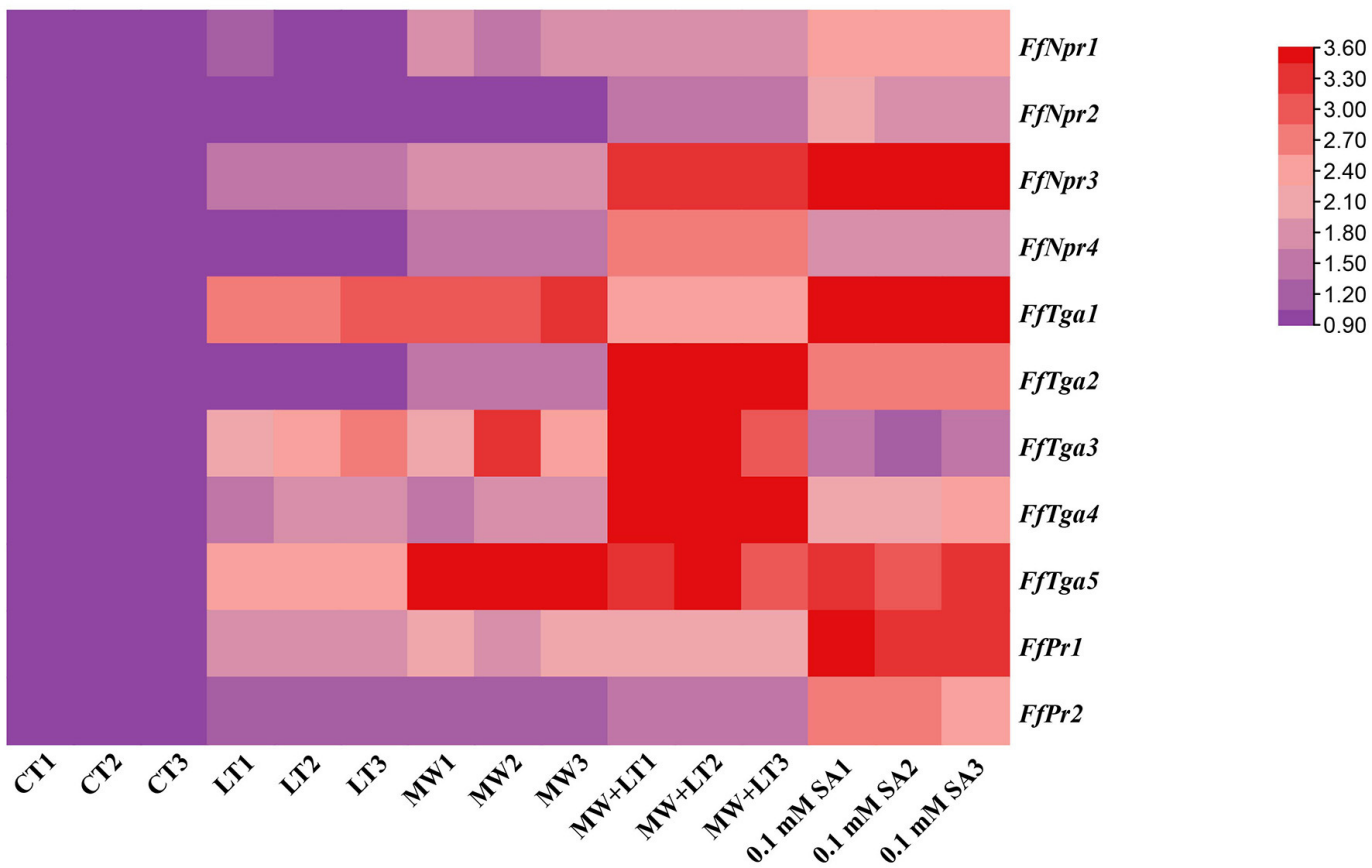
Heatmap of relative transcription levels of SA signaling pathway genes (*FfNpr, FfTga*, and *FfPr*) in *F. filiformis* under four treatments, namely LT, MW, MW + LT, and 0.1 mM exogenous SA. CT, Control (no treatment); LT, low temperature; MW, mechanical wound; MW + LT, the combination of mechanical wound and low temperature. The red depth indicates the higher relative expression.

The expression patterns of SA signal pathway genes (*FfNpr, FfTga*, and *FfPr*) in response to LT, MW, MW + LT, and 0.1 mM exogenous SA of SA pathway genes were performed by RT-qPCR. As shown in Figure 10, compared with the control (no treatment), the transcription levels of *FfNpr3* were significantly upregulated by ~1.6 to 5.7 times under LT, MW, MW + LT, and 0.1 mM SA treatments, while *FfNpr1/2/4* showed a significant increase in the transcription levels with varying degrees under only MW + LT and SA treatments, without differential expression under LT and MW. Five members of the *FfTga* family genes also showed differences in expression patterns under four treatments. The transcription levels of *FfTga1* and *FfTga5* were significantly upregulated by ~2.4 to 4.2 times and 2.5 to 8.5 times under four treatments, respectively. There was no significant difference in transcription levels of *FfTga2/4* under LT or MW and of *FfTga3* under 0.1 mM SA, compared with the control (no treatment). *FfPr1* showed a significantly upregulated expression with varying degrees (1.8 to 3.4 times) under four treatments, while *FfPr2* showed no significant difference in the transcription levels under LT, MW, and MW + LT treatments, except that there was an upregulated response expression (2.6 times) under 0.1 mM SA treatment.

## Discussion

Temperature change and external wound are the environmental factors that fungi are most susceptible to suffer from in nature. Low temperature and mechanical wound have been empirically used in production to stimulate the primordia formation of *F. filiformis*. In this study, LT, MW, and their combination MW + LT treatments were studied, analyzed their effect on time, quantity, and process of primordia formation of *F. filiformis* in detail, and also compared with a low concentration SA (0.1 mM SA) treatment. LT can stimulate the early formation of primordia but with a very small amount and a longtime span, maybe because the mycelia surface cannot develop into primordia at multiple nodes simultaneously only under the LT condition. On the contrary, MW can effectively stimulate many nodes resulted by cutting wound on every part of the mycelia surface to develop into a huge amount of primordia, which is of great significance to the production of *F. filiformis*. Therefore, the number of primordia under the wound treatment (MW and MW + LT) is tens or even hundreds of times more than that formed on the normal surface of the mycelia (without wound treatment). In plants, large amounts of intracellular SA hormone are rapidly produced after suffering from low temperature and tissue wound, and corresponding stress responses are triggered to activate systemic acquired resistance (Mercedes et al., 2003; Dong et al., 2014; Mostafa et al., 2022). In this study, the content of intracellular SA was increased significantly with varying degrees under three treatments (LT, MW, and MW + LT), and this leads to the point that there is a probable correlation between SA hormone and primordia formation. As shown in Figures 3B, 5, the content of SA under different treatments also generally showed a positive correlation with the amount of primordia formation. In addition, a low concentration of SA (0.1 mM SA) also significantly stimulated the formation of primordia in *F. filiformis*, and the formation time and developmental process were similar to the MW + LT treatment (Figure 3), further strengthening the inference that SA hormone was related to the formation of primordia.

As a hormone, SA plays a pivotal role in response to biotic and abiotic stresses (Horváth et al., 2007; Hayat et al., 2010). For example, *Arabidopsis* plants were infected by the pathogen, and SA levels were rapidly increased (O'Donnell et al., 2003; Love et al., 2005). In addition, when plants suffered from cold, drought, chilling, and wound, there was also a large secretion of pathogenesis-related proteins and hormones, including SA (Bertini et al., 2003; Seo et al., 2010). In the face of many external factors, plants respond to stress through SA signals and induce systemic acquired resistance (SAR). In fungi, when moderate stress (such as LT and MW) is applied to the physiologically mature mycelia, it also causes cells to make corresponding physiological changes and will also trigger the transformation of morphological development (when appropriate, it can turn to fruiting body formation) (Pleszczynska et al., 2013; Sakamoto, 2018). In other words, the fruiting body formation is also a protective way for fungal mycelia to cope with external stresses, which means that the formation of fruiting bodies initiates reproductive development to form the next generation of sexual spores. Therefore, the function of SA in regulating the growth and development of fruiting bodies can be manifested in fungi.

The specific function of SA depends on its activation of the downstream signaling pathway, to further regulate the expression of a series of related genes. Therefore, the identification and expression of SA downstream signaling pathway genes under various stress conditions are the main focus of this study. According to the basic model of the SA signaling pathway that has been constructed in plants, there are three main types of genes, such as *NPR, TGA*, and *PR* (Seyfferth and Tsuda, 2014; Han et al., 2022). In model organisms such as *Arabidopsis* and *N. tabacum*, the expression and function of different members of the *NPR* family have been well studied. NPR as a transcription co-activator mainly contains two protein–protein interaction domains: Ankyrin repeat domain and BTB/POZ domain (Wu et al., 2012). The BTB/POZ domain is beneficial to dimerize NPR, and the Ankyrin repeat domain interacts with the TGA of bZIP transcription factor (Rochon et al., 2006; Boyle et al., 2009). In this study, four members of the NPR family were identified in *F. filiformis*. Unlike plants, only FfNPR3 contains two conserved domains (ANK and BTB/POZ), while FfNPR1/2/4 contains only one of the two above conserved domains. Similar findings have been made in other fungi, according to the phylogenetic tree (Figure 6B). In combination with the transcriptional level, *FfNpr3* was also significantly upregulated in response to all four tested treatments, while *FfNpr1/2/4* showed no response expression for partial treatments in this study. Together with the obvious nuclear localization signaling in its promoter, *FfNpr3* plays a central role in the SA signaling pathway of *F. filiformis*, while the roles and specific functions of *FfNpr1/2/4* need to be further studied.

In plants, NPR proteins have been identified as core SA receptors, and all NPRs bind TGACG motif-binding (TGA) transcription factors which belong to the basic leucine zipper protein family (Kesarwani et al., 2007). In *Arabidopsis*, a total of 10 members of the TGA family are grouped into five clades, and three of them are known to interact with NPR. Clade II TGAs (TGA2, TGA5, and TGA6) have redundant functions in SA-induced pathogen resistance and controlling SAR (Zhang et al., 2003). Five members of the TGA family were identified in *F. filiformis* and were divided into three types based on gene structure. Clade I FfTGA (FfTGA3 and FfTGA4) contains no intron; Clade II FfTGA1 contains introns and multiple domains; Clade III FfTGA (FfTGA2 and FfTGA5) contains introns and only one domain, which is also different from plants.

In *Arabidopsis*, the NPR1 and NPR3/4 proteins have opposing roles in SA signaling: NPR1 activate, and NPR3/4 proteins repress, the SA-activated pathway, because SA binding to NPR1 induces NPR1-dependent target gene expression, whereas SA inhibits NPR3 and NPR4, which repress the expression of SA-induced genes (Ding et al., 2018). Due to the differences in the types and number of conserved domains of the NPR family between plants and fungi, *F. filiformis* FfNPR1/2/3/4 may not be the orthologous proteins of NPR1/2/3/4 in *Arabidopsis*. Therefore, there is also no direct evidence on whether there is a negative correlation between the expression of *FfNpr3* and *FfNpr1* in *F. filiformis*. Moreover, in this study, only a low concentration of SA (0.1 mM) was used for treatment. As shown clearly in Figure 1, a high concentration of SA (2 mM) can significantly inhibit the growth of the mycelia of *F. filiformis*, and the primordia will not be formed if the mycelia are not fully grown and physiologically mature in the culture bottle. Therefore, it is predictable that a high concentration of SA will not promote the fruiting body formation of *F. filiformis*.

**Figure 11:**
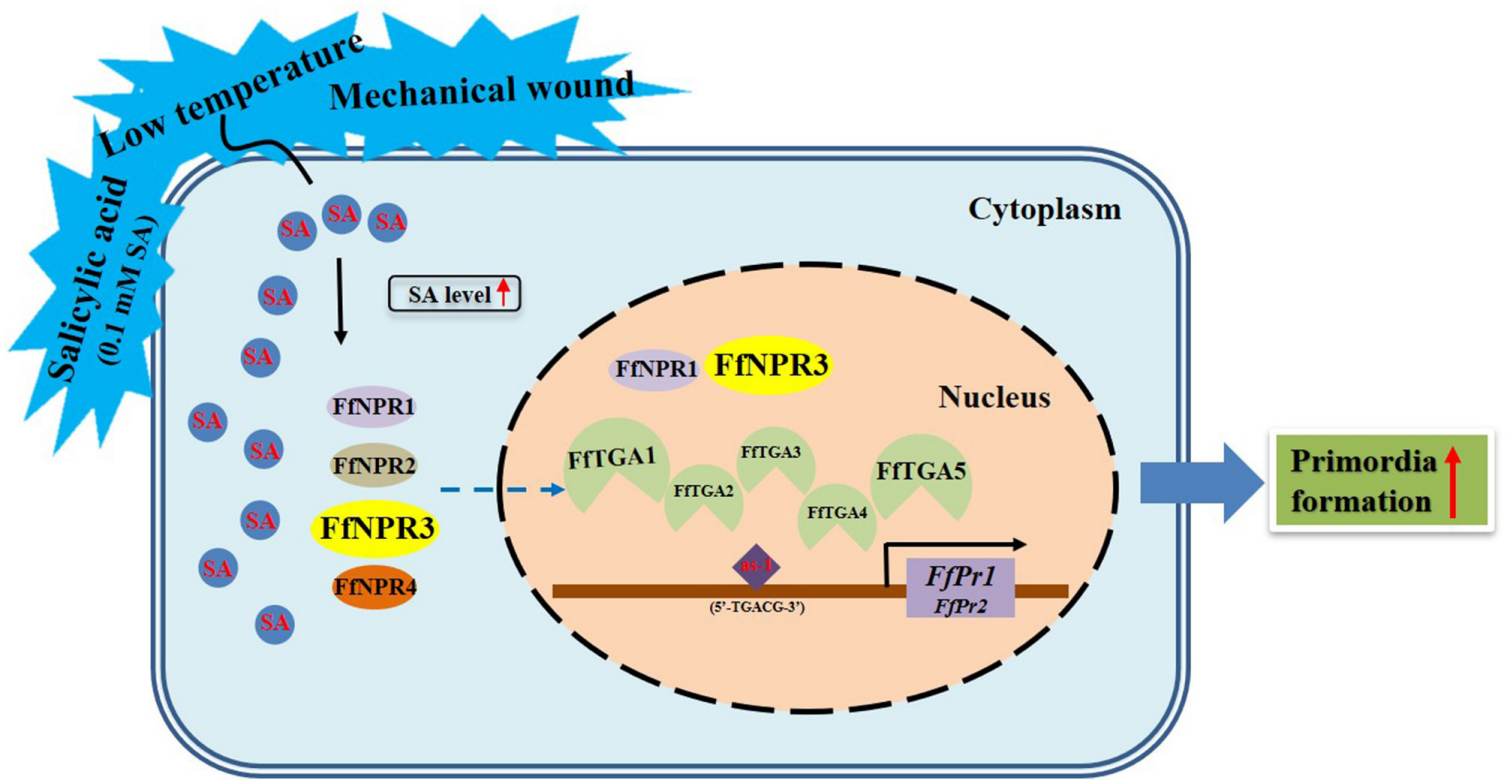
Model diagram of external factors including LT, MW, and 0.1 mM exogenous SA stimulating the SA signaling molecule as well as its downstream pathway genes and the formation of fruiting bodies in *F. filiformis*.

Endogenous SA accumulation stimulates the translocation of NPR into the nucleus, where it interacts with the TGA transcription factors and enhances the binding to the promoters of *PR* genes, thus ultimately activating the expression of *PR* genes (Chen et al., 2009). Both genes of *FfPr1* and *FfPr2* were found to contain as-1 motif sites, which is important for SA-inducible gene expression, and were significantly upregulated under four tested treatments. Together with the content changes of endogenous SA levels and the identification and expression patterns of SA signaling pathway genes under the four treatments, we summarize a draft model diagram of SA signal during the formation of fruiting bodies in *F. filiformis* (Figure 11). This study also implies that the function of SA hormones and the expression and mechanism of the SA signaling pathway in fungi may be complex and specific, which needs to be further studied under various environmental or stress conditions. Furthermore, the downstream target genes and metabolic pathways regulated by *PR* genes and associated with fruiting body formation in fungi, which also needs to be identified and detailed demonstrated in future studies.

## Data availability statement

The original contributions presented in the study are included in the article/Supplementary material, further inquiries can be directed to the corresponding author.

## Author contributions

ZL, JW, and YT conceived, designed the research, and wrote the manuscript. ZJ, HL, JH, and LG conducted the experiments. CY, LX, JZ, BX, and YT analyzed the data and revised the manuscript. All authors read and approved the manuscript.
