# Supplementary material for: Low temperature, mechanical wound, and exogenous salicylic acid (SA) can stimulate the SA signaling molecule as well as its downstream pathway and the formation of fruiting bodies in Flammulina filiformis
Source: Front Microbiol. 2023 Aug 22;14:1197498. doi: 10.3389/fmicb.2023.1197498 (PMC10477995; doi:10.3389/fmicb.2023.1197498)
Supplement: Supplementary file 1 [file Table_1.DOCX]

Supplementary Material

**Low temperature, mechanical wound and exogenous salicylic acid (SA) can stimulate the SA signaling molecule as well as its downstream pathway and the formation of fruiting bodies in** ***Flammulina filiformis***

**Ziyan Li^1,2,#^, Jin Wen^1,2,#^, Zhuohan Jing^1,2^, Hui Li^3^, Jiahua Huang^1,2^, Chengjin Yuan^1,2^, Lijun Xian^1,2^, Lingling Gao^1,2^, Jian Zhu^1,2^, Baogui Xie^2^, Yongxin Tao^1,2,*^**

^1^College of Horticulture, Fujian Agriculture and Forestry University, Fuzhou, 350002, Fujian, China

^2^Mycological Research Center, Fujian Agriculture and Forestry University, Fuzhou, 350002, Fujian, China

^3^Institute of Cash Crops, Hebei Academy of Agriculture and Forestry Sciences, Shijiazhuang 050051, China; lihuiviphappy@163.com

* Corresponding author: Yongxin Tao (Y. T)

To Corresponding author: College of Horticulture, Fujian Agriculture and Forestry University; No. 15 shangxiadian Road, Fuzhou 350002, Fujian, China

Telephone and Fax: 0086-0591-83789281

E-mail: taoyongxin@fafu.edu.cn

# Supplementary Figures and Tables

## Supplementary Tables

**Table S1.** Primers for RT-qPCR of salicylic acid signaling pathway genes in *Flammulina filiformis*.

| Gene name | Primer | Sequence (5′ to 3′) | Annealing  temperature  (°C) | Product size  (bp) |
| --- | --- | --- | --- | --- |
| *RNB* | *FfRnb*-qF | TTGTTCCTCTGTGTTCTCATCTC | 62 | 216 |
|  | *FfRnb-*qR | GACATCCTTCGTGGACCAATAG |  |  |
| *V-ATP* | *FfV-atp-*qF | GCAGAATGGTATGAGCGAGTAT | 62 | 173 |
|  | *FfV-atp-*qR | TCTGGGACAGCTTGAACATAAG |  |  |
| *β-TUB* | *Ffβ-tub-*qF | CAAATGCAGAACGTCCAGAAC | 62 | 242 |
|  | *Ffβ-tub-*qR | GTGAACTCCATCTCGTCCATAC |  |  |
| *FfNpr1* | *FfNpr1*-qF | CGCTCTAAGACTCGCATGAATA | 59 | 163 |
|  | *FfNpr1*-qR | TGTCGATGTAGCTGGACTTTAC |  |  |
| *FfNpr2* | *FfNpr2*-qF | GTAGGCTTCGTCAAACTCCTT | 57 | 172 |
|  | *FfNpr2-*qR | CTTGATTCAGCCGACCTCTATC |  |  |
| *FfNpr3* | *FfNpr3*-qF | GAAGTCACCCACGACAAGAAA | 59 | 196 |
|  | *FfNpr3*-qR | GCTACTGCTGTTGTCCATACTC |  |  |
| *FfNpr4* | *FfNpr4*-qF | CGCTTGGCTATCTCTACTCATC | 58 | 130 |
|  | *FfNpr4*-qR | CGACATTCTTCATAGGCGTACT |  |  |
| *FfTga1* | *FfTga1*-qF | GACGACGACGATGATGATGAA | 58 | 121 |
|  | *FfTga1*-qR | CCTCTGTCTCTGGTTTCTTCTG |  |  |
| *FfTga2* | *FfTga2*-qF | AGGATACCTCACCTCCAATCT | 57 | 116 |
|  | *FfTga2*-qR | CGTGAGAGTCGTTTACGTTAGG |  |  |
| *FfTga3* | *FfTga3*-qF | CTGACCAGGAGAAGATCGAATAC | 57 | 134 |
|  | *FfTga3*-qR | CCAGACCTCCTTCTCAATCATC |  |  |
| *FfTga4* | *FfTga4*-qF | GAGACTGCTCCAACGCTATAC | 57 | 144 |
|  | *FfTga4*-qR | GCCAGTAGGTACGCTTGATAG |  |  |
| *FfTga5* | *FfTga5*-qF | CGCAGATGACCTAGAAGTTGAG | 56 | 218 |
|  | *FfTga5*-qR | TACCCATACCCATTCCCATAGA |  |  |
| *FfPr1* | *FfPr1*-qF | GCCACACCTACTCCTTCAAATA | 55 | 193 |
|  | *FfPr1*-qR | CTCCACCTGAATGCTCAAAGA |  |  |
| *FfPr2* | *FfPr2*-qF | CCTGTCAGATCAGAGCTCATTC | 58 | 147 |
|  | *FfPr2*-qR | CATGCGACTAGAGTGGTATCTTC |  |  |

**Table S2.** Number of introns and exons of salicylic acid signaling pathway genes in *F. filiformis.*

| **Gene** | **Length of gene** | **Number of introns** | **Number of exons** |
| --- | --- | --- | --- |
| *FfNpr1* | 5981 bp | 12 | 13 |
| *FfNpr2* | 1291 bp | 10 | 11 |
| *FfNpr3* | 4426 bp | 10 | 11 |
| *FfNpr4* | 1310 bp | 6 | 7 |
| *FfTga1* | 1281 bp | 1 | 2 |
| *FfTga2* | 1980 bp | 5 | 6 |
| *FfTga3* | 597 bp | 0 | 1 |
| *FfTga4* | 879 bp | 0 | 1 |
| *FfTga5* | 1081 bp | 1 | 2 |
| *FfPr1* | 1081 bp | 3 | 4 |
| *FfPr2* | 647 bp | 2 | 3 |
